# Supplementary material for: β-Secretase-1: In Silico Drug Reposition for Alzheimer’s Disease
Source: Int J Mol Sci. 2023 May 3;24(9):8164. doi: 10.3390/ijms24098164 (PMC10179340; doi:10.3390/ijms24098164)
Supplement: Supplementary file 1 [file ijms-24-08164-s001.zip › ijms-2347264-supplementary.pdf]

Supplementary Material

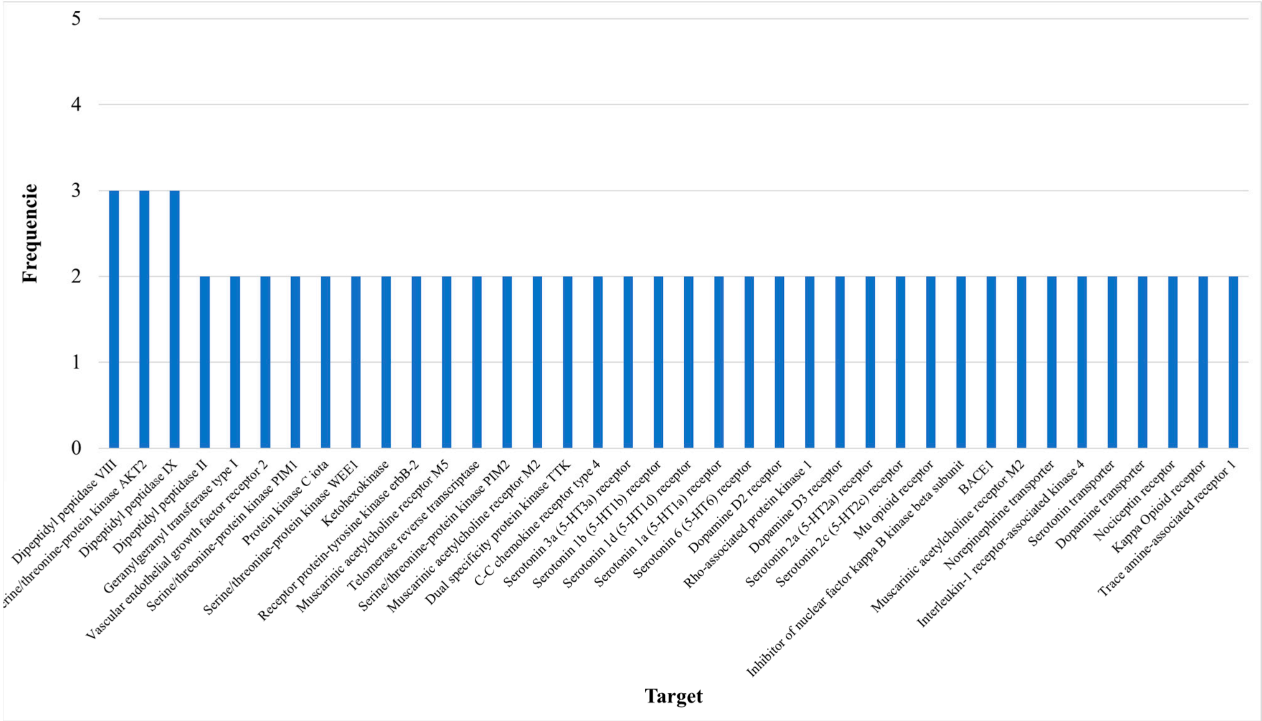

Figure S1. Frequency diagram by Swiss Target Prediction.

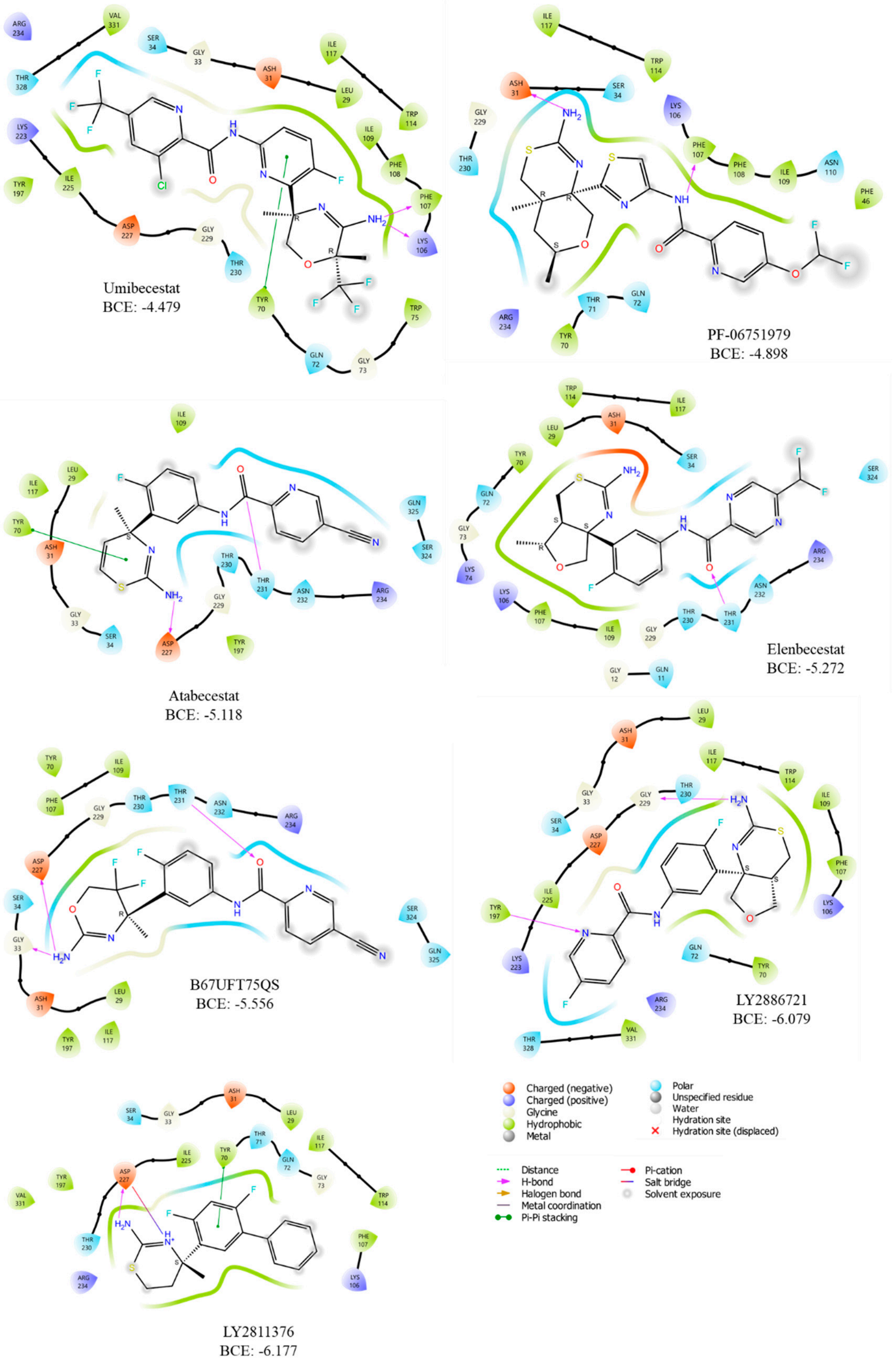

Figure S2. 2D interaction and Binding coupling energy of reference inhibitors in BACE-1.
